# Large-scale integrated analysis of ovarian cancer tumors and cell lines identifies an individualized gene expression signature for predicting response to platinum-based chemotherapy

**DOI:** 10.1038/s41419-019-1874-9

**Published:** 2019-09-10

**Authors:** Jie Sun, Siqi Bao, Dandan Xu, Yan Zhang, Jianzhong Su, Jiaqi Liu, Dapeng Hao, Meng Zhou

**Affiliations:** 1 School of Ophthalmology & Optometry and Eye Hospital, School of Biomedical Engineering, Wenzhou Medical University, Wenzhou, 325027 P. R. China; 2 Faculty of Sciences, Department of Biology, Harbin University, Harbin, 150081 P. R. China; 3 Department of Breast Surgical Oncology, National Cancer Center/National Clinical Research Center for Cancer/Cancer Hospital, Chinese Academy of Medical Sciences and Peking Union Medical College, Beijing, 100021 P. R. China; 4 Faculty of Health Sciences, University of Macau, Macau, 999078 P. R. China

**Keywords:** Tumour biomarkers, Predictive markers

## Abstract

Heterogeneity in chemotherapeutic response is directly associated with prognosis and disease recurrence in patients with ovarian cancer (OvCa). Despite the significant clinical need, a credible gene signature for predicting response to platinum-based chemotherapy and for guiding the selection of personalized chemotherapy regimens has not yet been identified. The present study used an integrated approach involving both OvCa tumors and cell lines to identify an individualized gene expression signature, denoted as IndividCRS, consisting of 16 robust chemotherapy-responsive genes for predicting intrinsic or acquired chemotherapy response in the meta-discovery dataset. The robust performance of this signature was subsequently validated in 25 independent tumor datasets comprising 2215 patients and one independent cell line dataset, across different technical platforms. The IndividCRS was significantly correlated with the response to platinum therapy and predicted the improved outcome. Moreover, the IndividCRS correlated with homologous recombination deficiency (HRD) and was also capable of discriminating HR-deficient tumors with or without platinum-sensitivity for guiding HRD-targeted clinical trials. Our results reveal the universality and simplicity of the IndividCRS as a promising individualized genomic tool to rapidly monitor response to chemotherapy and predict the outcome of patients with OvCa.

## Introduction

Ovarian cancer (OvCa) is one of the most common cancers of the female genital system and is the most lethal gynecological cancer, accounting for ~2.5 and 5% of female cancer occurrences and deaths, respectively^1^. Despite a decreasing trend in incidence and mortality rates over the past few years, patients with OvCa still face low 5-year relative survival rates of no more than 50% due to late-stage diagnoses^2^. The standard first-line treatment for newly diagnosed OvCa patients is aggressive cytoreductive surgery followed by platinum-based adjuvant chemotherapy^3^. Unfortunately, OvCa patients following first-line treatment experience heterogeneity in chemotherapeutic response. Almost 30% of patients do not respond to platinum-based chemotherapy and suffer long-term or even permanent side effects^4^. To date, there are no effective clinical measures to predict response to platinum-based chemotherapy. Thus, there is an urgent need to identify promising and individualized predictive molecular markers of response to platinum-based chemotherapy to optimize the use of chemotherapy for the treatment of patients with OvCa.

Advances in molecular “omics” strategies have revealed the significant genetic and molecular heterogeneity of OvCa that could influence the prognostic and chemotherapeutic response of patients^5–9^. These strategies also provide novel opportunities for identifying potential biomarkers for predicting patient outcome and chemotherapeutic response. Several studies have proposed several gene expression-based signatures for predicting chemotherapy response^4,8,10–16^. For example, by analyzing patients with serous OvCa from The Cancer Genome Atlas (TCGA) database, Kang et al.^4^ was the first to identify a DNA repair pathway-associated 23-gene set that predicts response to platinum therapy. Subsequently, Gonzalez Bosquet et al.^13^ developed a 422-gene signature model as a predictor of chemotherapeutic response. An additional study by Marchini et al.^11^ analyzed 23 tumor biopsies from patients with stage III-IV disease and identified an epithelial-mesenchymal transition (EMT)-related pathway signature associated with chemotherapy resistance. Unfortunately, none of these existing gene signatures have been widely adopted in clinical practice, which may be due, in part, to the use of only one dataset for discovery that does not account for biological heterogeneity, technical biases across different datasets, the large number of genes in a signature or impractical linear scoring modeling methods^17,18^. With the current availability of large-scale transcriptional profiles of OvCa tumors and cell lines, there is now an opportunity to identify and validate a robust and reproducible individualized gene signature for predicting response to platinum-based chemotherapy in OvCa.

In the present study, integrated transcriptional profiling analysis of multiple OvCa tumor and cell line datasets was performed to identify chemotherapy-responsive genes and develop an individualized gene expression signature that predicts intrinsic or acquired chemotherapy responsiveness. The robust performance of this signature was further validated in 25 independent tumor datasets comprising 2215 patients and one independent cell line dataset across different technical platforms.

## Materials and methods

### OvCa datasets and study design

The gene expression profiles and clinical characteristics of OvCa patients and cell lines were retrospectively collected from the Gene Expression Omnibus (GEO), The Cancer Genome Atlas (TCGA) network and the literature. A total of 29 public OvCa datasets were analyzed in this study, including three cell line datasets and 26 patient datasets derived from 12 different microarray platforms and two RNA-Seq platforms. Of these, 11 OvCa datasets included chemotherapeutic response information and 21 datasets included overall survival (OS) information. Detailed information about the 29 OvCa datasets can be found in Supplementary Table 1.

Out of the 11 OvCa datasets with chemotherapeutic response information, the following four large datasets: Dressman, Patch and TCGA (all patient datasets) and the GSE47856 cell line dataset, were selected as a meta-discovery dataset for identifying robust chemotherapy-responsive genes based on the following criteria: (i) dataset with chemotherapeutic response information; (ii) patient dataset with overall survival information; (iii) dataset with larger samples. The remaining 25 individual datasets were used as testing datasets for independent validation.

### Pre-processing and analysis of gene expression profiles

Raw data from the microarray datasets were derived from Affymetrix HG-U133A and HG-U133_Plus_2 platforms and were downloaded and processed using the Robust Multichip Average algorithm for background correction, quantile normalization and log2-transformation. Processed data from other platforms provided by authors or other available databases were also used. When multiple probes were mapped to the same gene, the microarray probe set that was the most highly expressed was used to represent the expression value of that gene^19^. Differentially expressed genes between resistant samples and sensitive samples were determined using R package “limma” for microarray datasets and R package “edgeR” for RNA-Seq datasets.

### Construction of an individualized gene signature for predicting response to chemotherapy

Differentially expressed genes identified in at least three out of the four meta-discovery datasets were considered as robust chemotherapy-responsive genes. Individualized chemotherapy response scores (IndividCRS) was then generated and used to predict chemotherapeutic response using the T statistic of a two-sided *t*-test for each sample by comparing the average expression level of the six chemotherapy-sensitive genes with the average of 10 chemotherapy-resistant genes according to previous study [5] as follows:

$${\mathrm{IndividCRS}} = \frac{{\overline {X_{CS}} - \overline {X_{{\mathrm{CR}}}} }}{{\sqrt {\frac{{\left( {n_{{\mathrm{CS}}} - 1} \right)S_{CS}^2 + \left( {n_{{\mathrm{CR}}} - 1} \right)S_{{\mathrm{CR}}}^2}}{{n_{{\mathrm{CS}}} + n_{{\mathrm{CR}}} - 2}}\left( {\frac{1}{{n_{{\mathrm{CS}}}}} + \frac{1}{{n_{{\mathrm{CR}}}}}} \right)} }}$$

Where *n*_cs_ is the number of chemotherapy-sensitive genes and *n*_CR_ is the number of chemotherapy-resistant genes. $S_{{\mathrm{CS}}}^2$ is the expression variance of chemotherapy-sensitive genes and $S_{{\mathrm{CR}}}^2$ is the expression variance of chemotherapy-resistant genes. An IndividCRS of >0 meant that the 6 chemotherapy-sensitive genes were overexpressed, while the 10 chemotherapy-resistant genes were underexpressed, whereas an IndividCRS of <0 meant that the opposite was true. Therefore, IndividCRS would be positively correlated with chemotherapy response, serving as a potential indicator of chemotherapy response

### Statistical analysis

Comparisons between groups were performed using the Wilcoxon rank-sum test, unpaired or paired *t*-tests as indicated. Differences in survival were determined using Kaplan–Meier curves and the log-rank test. *P* < 0.05 was considered to indicate a statistically significant difference and all tests were two-sided. All statistical analyses were performed using R (version 3.4.0).

## Results

### Development of an individualized gene signature for predicting response to chemotherapy

**Fig. 1:**
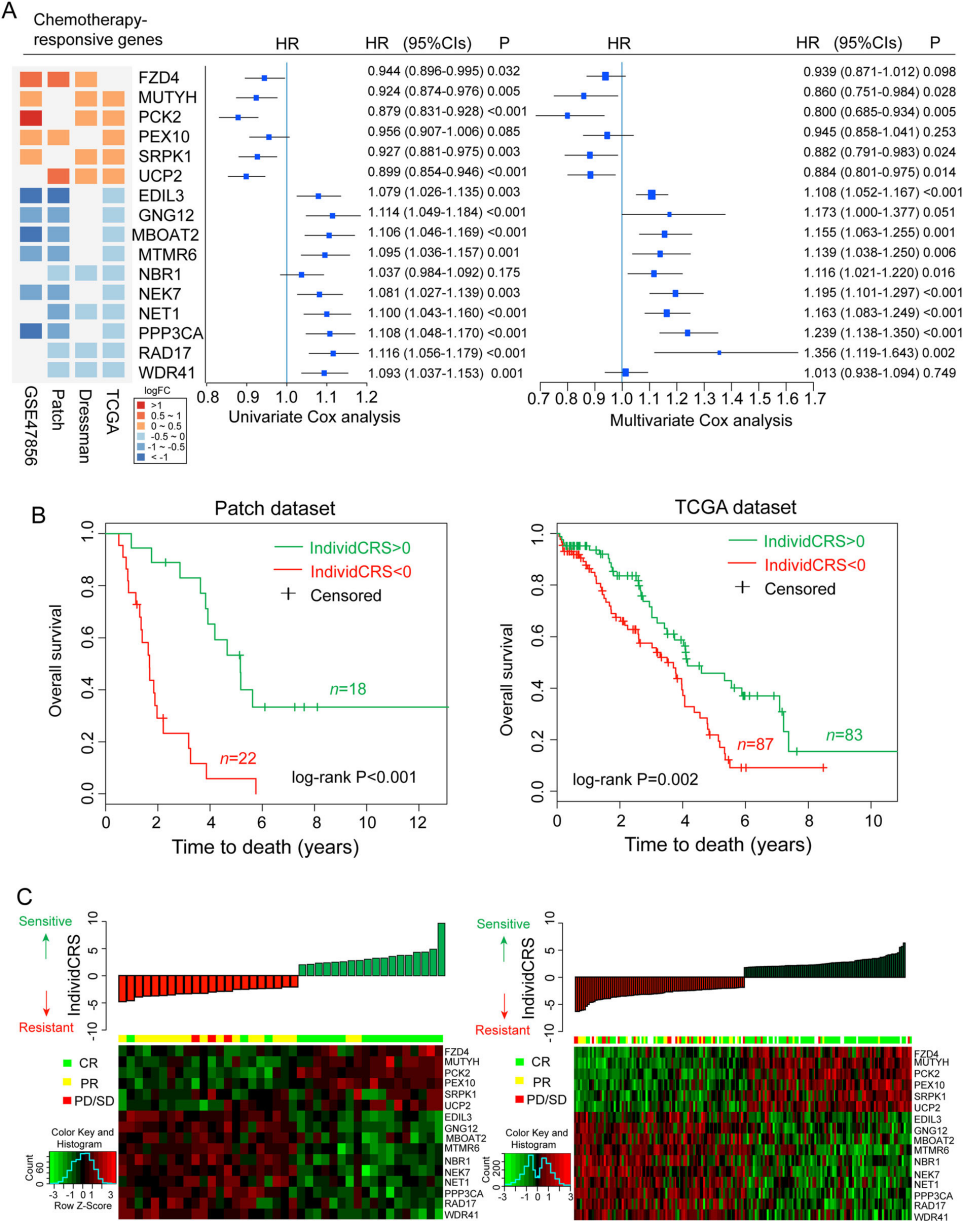
Development of a 16-gene signature. **a** Forest plot illustrating the Hazard ratios (HRs) of fixed-effect meta-analysis summary result for each of the 16 chemotherapy-responsive genes. The HR shown for each gene is leveraged from univariate and multivariate Cox-PH analysis across 21 ovarian cancer cohorts. Colored rectangles on the left indicate the logFC of gene expression comparing chemo-sensitive to chemo-resistant tumors. **b** Kaplan–Meier curves of OS for patients divided by the IndividCRS in the Patch and TCGA datasets. Significance was determined using the log-rank test. **c** Distribution of the IndividCRS and expression heatmap of 16 chemotherapy-responsive genes in the Patch and TCGA datasets. HR, hazard ratio; OS, overall survival; IndividCRS, individual chemotherapeutic response scores; TCGA, The Cancer Genome Atlas Network; CR: Complete response; PR: partial response; PD: progression disease; SD: stable disease

In clinical studies, response to chemotherapy is estimated based on the progression of disease during therapy. However, in addition to the effect of chemotherapeutic agents, disease progression depends on a number of factors, such as the immune microenvironment and the angiogenesis of tumors. Therefore, multiple patient cohorts and cell line datasets were analyzed in the present study to identify common chemotherapy-responsive genes. Cross-validation was used to prevent over-fitting by performing meta-discovery using 754 patients from three cohort studies and 171 transcriptomes from OvCa cell lines representing the largest datasets with detailed clinical information and the largest cisplatin-treated cell line dataset, respectively. The results were then validated in additional patient cohorts and cell line datasets. Differential expression analysis between resistant and sensitive samples in the meta-discovery dataset was performed, and the top 1000 differentially expressed genes were selected as candidate chemotherapy-responsive genes; this was because the significance is affected by the number and the homogeneity of samples. The overlap of these candidate chemotherapy-responsive genes was observed to be poor among the four datasets in the meta-discovery dataset (Fig. S1). Therefore, chemotherapy-responsive genes that appeared in at least three of the four datasets in the meta-discovery dataset were selected as candidate robust chemotherapy-responsive genes. Finally, the following 16 robust chemotherapy-responsive genes were identified, which included six chemotherapy-sensitive genes: *(FZD4*, *MUTYH*, *PCK2, PEX10, SRPK1*, *UCP2*), as well as the following 10 chemotherapy-resistant genes: (*EDIL3*, *GNG12, MBOAT2, MTMR6, NBR1, NEK7, NET1, PPP3CA*, *RAD17 WDR41*) (Fig. 1a). To examine the whether the prognosis performance of 16 chemotherapy-responsive genes are independent of clinical variables including age, grade and stage, we performed multivariate Cox analysis in each of the OS-containing patient datasets, followed by a meta-analysis of all 21 patient datasets. The results of the meta-analysis suggested that 14 of 16 chemotherapy-responsive genes still are significant or marginally significant associated with OS after adjusting for other clinical variables (Fig. 1a).

Based on these observations, an individualized gene expression signature was then developed, denoted as IndividCRS, which consisted of 16 chemotherapy-responsive genes as a predictor of chemotherapeutic response (see Materials and methods). The predictive value of the IndividCRS was first evaluated in the Patch and TCGA datasets. The IndividCRS significantly stratified patients into low- versus high-risk groups in terms of OS. Patients with significantly high IndividCRS scores (>0) had a more favorable prognosis and higher chemotherapy sensitivity than patients with significantly lower IndividCRS scores (<0) (Patch dataset, log-rank test *P* < 0.001; TCGA dataset, log-rank test *P* = 0.002; Fig. 1b). As expected, overexpression of six chemotherapy-sensitive genes was associated with higher chemotherapeutic sensitivity, whereas overexpression of 10 chemotherapy-resistant genes was associated with higher chemotherapeutic resistance (Fig. 1c). To further examine the relationship between the IndividCRS and the likelihood of complete response (CR), the IndividCRS were divided into 10 equal intervals in the TCGA datasets and the percentage of patients achieving a CR against each interval of increasing IndividCRS was plotted. Similar results were also observed for all patients in the Patch dataset and TCGA dataset (Fig. S2). The results indicated that the probability of patients achieving CR was significantly correlated with the IndividCRS (Pearson correlation coefficient [*r*^2^] = 0.88, *P* < 0.001; Fig. S3). This demonstrated the predictive capacity of the IndividCRS for response to platinum-based chemotherapy.

### Validation of the IndividCRS in independent cell lines and patient samples

**Fig. 2:**
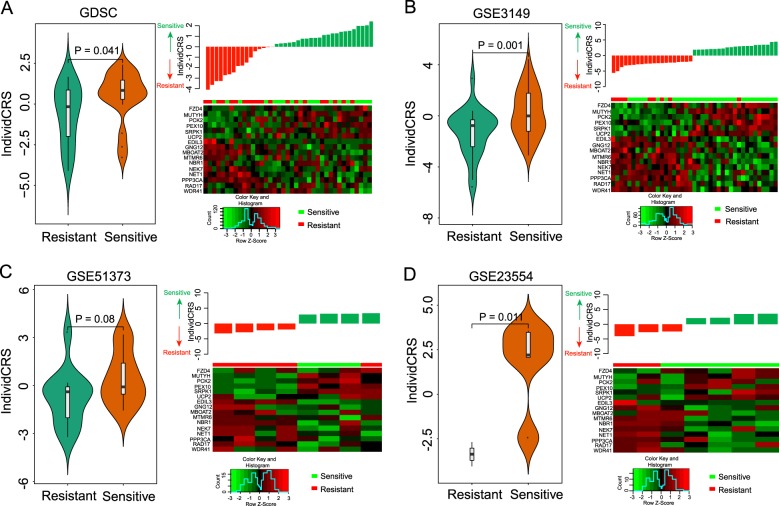
Association between the IndividCRS and chemotherapy response in four independent datasets. Violin Plot illustrating the distribution and probability density of the IndividCRS, and the distribution of the IndividCRS and expression heatmap of 16 chemotherapy-responsive genes in the **a** GDSC dataset, **b** GSE3149 dataset, **c** GSE51373 dataset, and **d** GSE23554 dataset. Patients with chemotherapy sensitivity exhibited higher IndividCRS than those with chemotherapy resistance. Significance was determined using an unpaired *t*-test. IndividCRS, individual chemotherapeutic response scores

To further ensure the validity of the IndividCRS, four independent dataset IndividCRS, including one cell line dataset (GDSC dataset) and three patient datasets (GSE3149, GSE51373, and GSE23554), were computed and analyzed with drug treatment information. For the cell line dataset, OvCa cell lines treated with cisplatin were divided into resistant and sensitive cell line groups using the median GI50 values. As shown in Fig. 2a, significantly higher IndividCRS were observed for sensitive cell lines when compared with resistant cell lines (unpaired *t*-test, *P* = 0.041). Furthermore, six chemotherapy-sensitive genes were observed to be overexpressed and the expression of 10 chemotherapy-resistant genes was reduced in sensitive cell lines. An inverse trend was observed in the resistant cell lines.

In the three patient datasets, a similar association between the IndividCRS and response to chemotherapy was observed. Patients that were sensitive to chemotherapy were observed to have a higher IndividCRS than patients with chemotherapy resistance (unpaired *t*-test; *P* = 0.001 for GSE3149; *P* = 0.08 for GSE51373; *P* = 0.011 for GSE23554; Fig. 2b–d). These results suggested that a high IndividCRS (>0) was associated with sensitivity to chemotherapy and a low IndividCRS (<0) was associated with chemotherapy resistance. Furthermore, patients that were sensitive to chemotherapy were more likely to express high levels of six chemotherapy-sensitive genes, whereas patients with chemotherapy resistance were more likely to express high levels of 10 chemotherapy-resistant genes, which is similar to the results observed in the meta-discovery dataset (Fig. 2b–d). These independent results, therefore, demonstrate that the IndividCRS may present an effective measure of chemotherapeutic response, as it is able to distinguish between sensitivity and resistance to chemotherapy in OvCa patients.

### Correlation between the IndividCRS and HRD

**Fig. 3:**
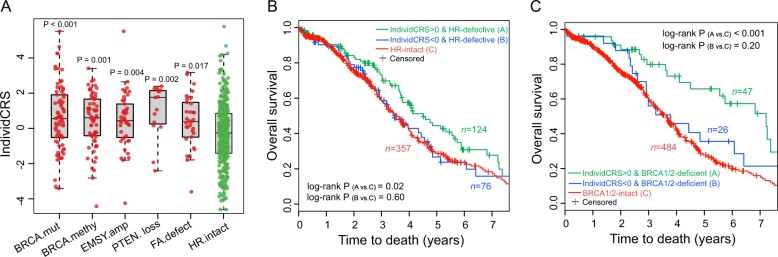
Association between the IndividCRS and HRD. **a** Pairwise comparison of the IndividCRS between HR-intact tumors and HR-defective tumors including those with a BRCA mutation, BRCA methylation, EMSY amplification, and defects in PTEN and FA genes. Significance was determined using the Wilcoxon rank-sum test. **b** Kaplan–Meier curves of OS for high IndividCRS HR-defective tumors and low IndividCRS HR-defective tumors versus HR-intact tumors. The high IndividCRS group of HR-defective tumors showed significantly improved OS, whereas low IndividCRS group of HR-defective tumors showed no significant difference in OS compared with HR-intact tumors. Significance was determined using the log-rank test. **c** Kaplan–Meier curves of OS for high IndividCRS BRCA-deficient tumors and low IndividCRS BRCA-deficient tumors versus BRCA1/2-intact tumors. The high IndividCRS group of BRCA-deficient tumors showed significantly improved OS, whereas low IndividCRS group of BRCA-deficient tumors showed no significant difference in OS compared with BRCA1/2-intact tumors. Significance was determined using the log-rank test. IndividCRS, individual chemotherapeutic response scores; HRD, homologous recombination deficiency; PTEN, phosphatase and tensin homolog; FA, Fanconi anemia; OS, overall survival

Recent studies indicate that the enhanced sensitivity of OvCa patients to platinum-based chemotherapy could largely be explained by HRD^20–22^. In the present study, the TCGA dataset (*n* = 557), that included patients treated with platinum-based chemotherapy, also provided DNA sequencing and methylation data. In this dataset, we identified 200 patients with BRCA mutations, BRCA methylation, defects of PTEN, defects of FA (Fanconi anemia) genes, Ras-related associated with diabetes (RAD) genes, and ATM (ataxia telangiectasia) genes (i.e., ATM, ataxia telangiectasia and Rad3-related (ATR), and CHEK1/2), as well as the amplification of EMSY as HR-defective tumors^21^. To investigate the relationship between the IndividCRS and HR deficiency, the distribution of IndividCRS between HR-defective tumors and HR-intact tumors was examined. A significantly higher IndividCRS in HR-defective tumors, when compared with HR-intact tumors, was observed (Fig. 3a). A total of 557 OvCa patients from the TCGA dataset were then divided into the following three groups: The HR-intact group, the high IndividCRS HR-defective group and the low IndividCRS HR-defective group. Pairwise comparisons of OS between the groups were performed and the results demonstrated that the high IndividCRS HR-defective group had a significantly better OS rate when compared with patients in the HR-intact group (log-rank test, *P* = 0.02; Fig. 3b). However, no significant difference in OS between the HR-intact group and the low IndividCRS HR-defective group was observed (log-rank test, *P* = 0.60; Fig. 3b). These results suggested that the IndividCRS are not only correlated with HRD, but can also identify a subset of HR-defective tumors with resistance to platinum-based chemotherapy. The association between BRCA1/2-deficient tumors and OS in OvCa was then analyzed using the IndividCRS. The results shown in Fig. 3c demonstrated that BRCA-deficient patients with high IndividCRS exhibit significantly better survival rates when compared with BRCA1/2-intact cases (log-rank test, *P* < 0.001). By contrast, BRCA-deficient patients with low IndividCRS demonstrated no significant difference in OS when compared with BRCA1/2-intact cases (log-rank test, *P* = 0.20; Fig. 3c). This implied that the IndividCRS could discriminate BRCA1/2-deficient tumors with various survival outcomes and distinct responses to platinum-based chemotherapy.

### Analysis of enhanced resistance in samples treated with multiple cycles of chemotherapy

**Fig. 4:**
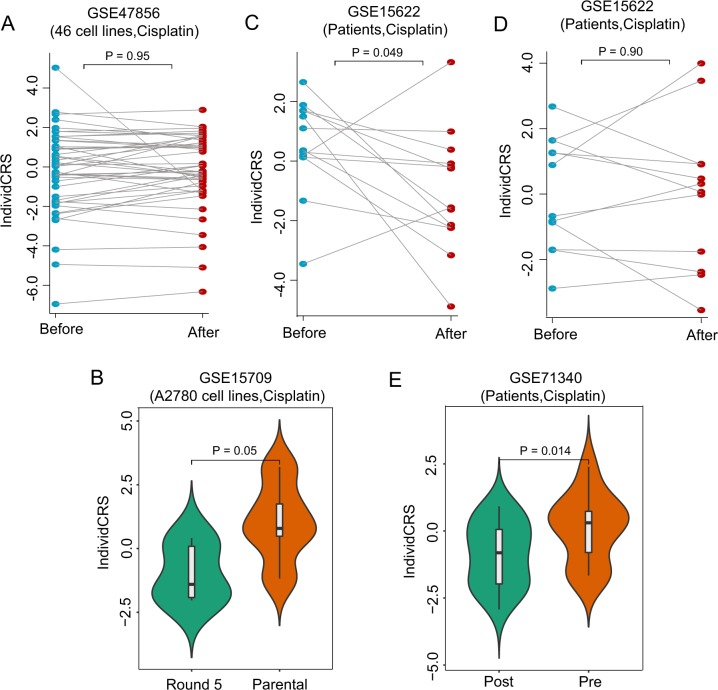
Association between the IndividCRS and enhanced resistance in samples treated with multiple cycles of chemotherapy. **a** Pairwise comparison of the IndividCRS in 46 cell lines before and after 48 h treatment with cisplatin. Significance was determined using a paired *t*-test. **b** Violin Plot illustrating the distribution and probability density of the IndividCRS in samples before and after five rounds of cisplatin treatment. Significance was determined using a paired *t*-test. **c** Pairwise comparison of the IndividCRS in patients before and after three cycles of carboplatin treatment. Significance was determined using a paired *t*-test. **d** Pairwise comparison of the IndividCRS in samples before and after three cycles of paclitaxel treatment. Significance was determined using paired *t*-test. **e** Pairwise comparison of the IndividCRS in samples post- and pre-chemotherapy. Significance was determined using an unpaired *t*-test. IndividCRS, individual chemotherapeutic response scores

To examine whether resistance to chemotherapy changes with drug treatment duration, the GSE47856 dataset was first used to evaluate changes in resistance over a short time-frame because this dataset provided expression profiles of 46 cell lines before and after 48 h treatment with cisplatin. By performing pairwise comparisons for the IndividCRS in 46 cell lines, no significant difference in the IndividCRS between cell lines before and after 48 h treatment with cisplatin was observed (paired *t*-test, *P* = 0.95; Fig. 4a). This suggests that cell lines do not acquire resistance after short-term drug treatment and the IndividCRS may also not be affected by short-term drug treatment.

Changes in chemotherapy resistance and IndividCRS before and after long-term platinum-based chemotherapy were then investigated in three patient datasets. In the GSE15709 dataset, platinum-sensitive A2780 OvCa cells were treated with incrementally increasing doses of cisplatin^23^. As shown in Fig. 4b, platinum-sensitive A2780 OvCa cells treated with five rounds of cisplatin exhibited a marked decrease in IndividCRS with increasing cisplatin resistance (paired *t*-test, *P* = 0.05). The GSE15622 dataset consisted of a mixed patient population treated with three cycles of either carboplatin or paclitaxel. The comparative analyses were repeated and a statistically significant difference in IndividCRS in patients before and after three cycles of carboplatin treatment was observed (paired *t*-test, *P* = 0.049; Fig. 4c). However, it is important to note that statistically significant differences in IndividCRS were not detected in patients before and after three cycles of paclitaxel treatment (paired *t*-test, *P* = 0.9; Fig. 4d), indicating that the IndividCRS is a specific predictor of response to platinum-based chemotherapy. A significant difference in IndividCRS was also observed in the GSE71340 dataset. As shown in Fig. 4e, the IndividCRS of pre-chemotherapy patients was significantly higher than that of post-chemotherapy patients (unpaired *t*-test, *P* = 0.014).

### Association between platinum resistance and EMT

**Fig. 5:**
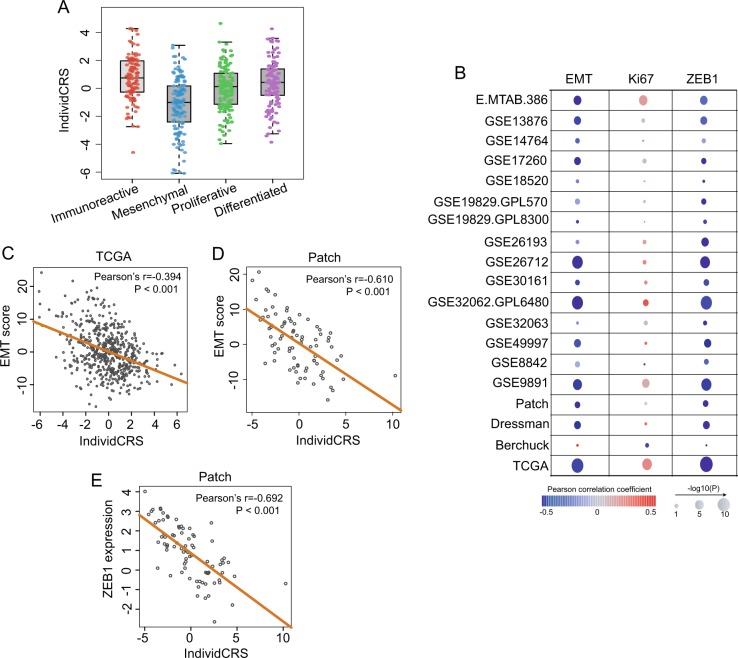
Association between platinum resistance and EMT. **a** Pairwise comparison of the IndividCRS in four OvCa subtypes, including immunoreactive, differentiated, proliferative and mesenchymal. Significance was determined using the Wilcoxon rank-sum test. **b** Bubble plot showing the correlation between the IndividCRS and EMT score, ZEB1 and Ki67 across 19 patient datasets. The size of the circles proportional to the significance of the correlation and the color of the border indicates Pearson correlation coefficients. Correlation of the IndividCRS with EMT score in the **c** TCGA dataset and **d** Patch dataset, and **e** ZEB1 in the Patch dataset. The orange line represents the line of linear regression through the data points. EMT, epithelial-mesenchymal transition; IndividCRS, individual chemotherapeutic response scores; OvCa, ovarian cancer; ZEB1, zinc finger E-box binding homeobox 1

Conflicting results regarding the role of EMT in chemotherapeutic response have been reported^24^. Therefore, to assess the contribution of EMT in resistance to platinum-based chemotherapy, the distribution of the IndividCRS were first analyzed and compared in four OvCa subtypes identified in the TCGA dataset, including immunoreactive, differentiated, proliferative and mesenchymal. As shown in Fig. 5a, the IndividCRS of patients with tumors of the mesenchymal subtype was significantly lower than that of the remaining three subtypes (Wilcoxon rank-sum test, *P* < 0.001; Fig. 5a), indicating that mesenchymal subtypes exhibited particularly high platinum-based chemoresistance.

A compendium of 145 epithelial marker genes and 170 mesenchymal marker genes were then obtained from the study by Tan et al.^25^ and an EMT score was derived using the t-test statistic generated by comparing the normalized expression of 170 mesenchymal marker genes with 145 epithelial marker genes. An EMT score of >0 indicated a mesenchymal-like phenotype and an EMT score of <0 indicated an epithelial-like phenotype. The EMT scores for OvCa patient datasets were calculated and a strong negative correlation between the EMT score and the IndividCRS was observed (Fig. 5b). For example, Pearson correlation coefficient analysis of the EMT score and the IndividCRS was −0.394 (*P* < 0.001) and −0.61 (*P* < 0.001) in the TCGA and Patch datasets, respectively (Fig. 5c, d). It has been reported that the zinc finger E-box binding homeobox 1 (ZEB1) transcription factor is a key driver of EMT and is involved in resistance to cancer therapy^26^. Therefore, associations between ZEB1 expression and the IndividCRS for OvCa patient datasets were analyzed in the present study. The results demonstrated a strong negative correlation between ZEB1 expression and the IndividCRS (Pearson correlation coefficient, *r*^2^ = −0.692, *P* < 0.001; Fig. 5e).

Previous studies have demonstrated that DNA replication is increased during cell proliferation, and an increased number of mutations are introduced by platinum-based agents^27,28^. Therefore, the association between IndividCRS and the Ki67 gene, which is a crucial marker of cellular proliferation, was examined in the present study^29^. The expression of Ki67 was positively correlated with the IndividCRS except in the Berchuck dataset (Fig. 5b), thus indicating that a positive correlation between cellular proliferation and sensitivity to platinum-based agents exists.

### Association of the IndividCRS with clinical outcome

**Fig. 6:**
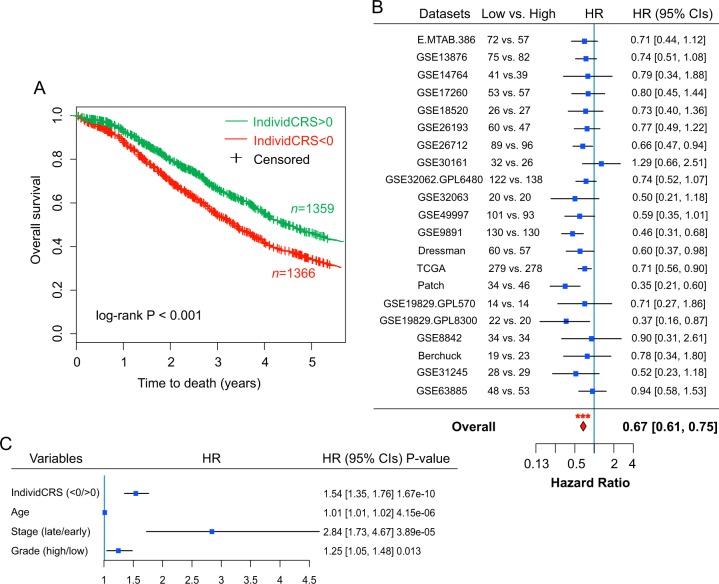
Prognostic value of the IndividCRS in predicting clinical outcome. **a**, **b** Kaplan–Meier curves of OS for patients in low IndividCRS and high IndividCRS subgroups. Significance was determined using the log-rank test. **b** Forest plot illustrating the HRs of survival analyses of low-IndividCRS group versus high-IndividCRS in 21 patient datasets. Significance was determined using the Cox proportional hazards model. The red diamond indicates the fixed-effects meta-analysis summary of HRs over 21 datasets. **c** Multivariable analyses using Cox proportional hazards models were performed for all patients in 21 datasets. IndividCRS, individual chemotherapeutic response scores; OS, overall survival; HR, hazard ratio, CI, confidence interval

The ability of the IndividCRS to predict clinical outcome was then examined by integrating 2725 patients from the 21 datasets together (except for the GSE51088 dataset, as only nine genes of the IndividCRS were covered in this dataset). Patients were divided into two groups based on their IndividCRS; patients with high risk (IndividCRS < 0) and patients with low risk (IndividCRS > 0). Survival analysis demonstrated that the two groups were associated with significantly different outcomes, with the high-risk group experiencing shorter OS when compared with the low-risk group (*n* = 1359 versus 1366, high versus low IndividCRS, HR = 0.71, 95% CI = 0.64–0.79, *P* < 0.001) (Fig. 6a). To avoid cross-dataset bias, survival analysis for each dataset was repeated independently, which was followed by a meta-analysis of 21 datasets to determine the overall prognostic value of the IndividCRS. Results from the meta-analysis demonstrated a strong prognostic value of the IndividCRS for OS (HR = 0.67, 95% CI = 0.61–0.75, *P* < 0.001; Fig. 6b). Furthermore, the IndividCRS still maintained a significant correlation with OS when adjusted by clinical factors (age, stage and grade) in multivariable analyses (HR = 1.54, 95% CI = 1.35–1.76, *P* < 0.001; Fig. 6c). We also examine the associations of IndividCRS with stage and grade, and found that there is no significant difference in IndividCRS between late-stage and early-stage patient groups, but the IndividCRS is significantly higher in the high-grade patient group than in low-grade group (Fig. S4) despite the fact that low-grade patients tend to have better outcome compared to high-grade patients. These observations indicated that the association of IndividCRS with chemotherapy response did not dependent on stage and grade. We also performed stratified analysis to examine whether the IndividCRS has prognostic value within the same clinical grade. Survival analysis suggested that the IndividCRS could classify low-grade patients into high-risk and low-risk groups. Similar prognostic value of the IndividCRS was also observed for high-grade patients (Figure S5). These results suggest that the IndividCRS may be an independent predictive factor in distinguishing worse versus improved survival outcomes in patients who did or did not receive platinum-based chemotherapy.

## Discussion

By combining gene expression profiles and chemotherapeutic response information from multiple patients and cell line datasets, the present study developed a gene expression signature comprising 16 chemotherapy-responsive genes, which could predict individual responses to platinum-based chemotherapy for patients with OvCa. Although platinum-based adjuvant chemotherapy is the first-line treatment and is associated with high response rates in OvCa patients, ~30% of patients have been found to develop chemoresistance following multiple cycles of chemotherapy, and ~50% of patients with CR relapse within a few years^4,18^. Therefore, identifying predictive markers of response to platinum-based chemotherapy is an ongoing focus of research despite continuous efforts have been made during the past number of years. A recent review revealed that the gene signatures identified by previous studies are very dissimilar, and the vast majority of genes were identified using only one study^18^. In contrast to previous studies, where informative genes were identified based solely on a single dataset and did not take into consideration the heterogeneity of patient cohorts, the present study integrated multiple patient and cell line datasets with chemotherapy treatment to identify the most commonly differentially expressed genes, which were demonstrated as reliable chemotherapy-responsive genes. These chemotherapy-responsive genes include genes involved in the repair of DNA damage (*MUTYH*, *NBR1*, *NEK7*, *NET1*, and *RAD17*), and genes previously reported to be associated with the response to chemotherapy (*FZD4*, *SRPK1* and *UCP2*)^30–32^.

To accelerate the clinical application of our findings, the 16 reliable chemotherapy-responsive genes were integrated to form a gene expression signature (denoted IndividCRS) by comparing expression differences between chemotherapy-sensitive and chemotherapy-resistant genes. Unlike previous linear scoring models, this signature design allowed IndividCRS to have a natural cutoff (IndividCRS = 0) that divided sensitive and resistant tumors. This was associated with statistical significance and eliminated the need for the pre-collection of samples for data normalization and the establishment of trained risk score thresholds. Moreover, the predictive value of the IndividCRS was validated in multiple independent OvCa datasets derived from different laboratories that used different platforms. This demonstrated that the IndividCRS is a robust tool that may allow for the individualized and single-sample prediction of response to platinum-based chemotherapy before initiation of first-line therapy in clinical practice.

Increasing evidence suggests that tumors with HR deficiency are more likely to be highly sensitive to platinum-based chemotherapy^20–22^. Detection of HR deficiency is a widely accepted strategy to identify platinum-sensitive tumors. However, it is worth noting that not all HRD tumors, including BRCA1/2-mutated tumors, are sensitive to platinum agents. Therefore, the relationship between HR deficiency and IndividCRS was examined in the current study, and a potential association between HR deficiency and IndividCRS was identified, indicating that the IndividCRS may be used as an indicator of HR deficiency which need be further investigated using functional experiments. In addition, the IndividCRS can further discriminate between chemoresistance and chemosensitivity in HR-deficient tumors, which may provide valuable information for guiding HRD-targeted clinical trials. Conflicting results have been reported regarding the outcome of OvCa patients harboring BRCA1/2 mutations. A number of studies have demonstrated that not all OvCa patients harboring BRCA1/2 mutations have significantly more favorable outcome than patients with wild-type BRCA, and some patients may even develop platinum resistance^33–35^. By applying the IndividCRS to BRCA-deficient patients in the present study, a significant difference in OS between BRCA-deficient patients with high IndividCRS and those with low IndividCRS was observed, suggesting that BRCA-deficient patients could be further discriminated by the IndividCRS. Therefore, the IndividCRS may have important clinical applications in identifying BRCA-deficient patients with unfavorable outcomes and allow redirection to more appropriate therapies in time.

Despite previous studies claiming that the existing signatures are able to predict survival or response to initial chemotherapy in OvCa patients, the majority of OvCa patients that initially respond to chemotherapy treatment develop the recurrent disease due to acquired resistance to platinum-based chemotherapy after undergoing multiple cycles of chemotherapy^36^. Therefore, the present study examined the relationship between changes in chemotherapy resistance and the IndividCRS during and after drug treatment. The results demonstrated that the IndividCRS reflected the dynamics of resistance during the treatment. Furthermore, the IndividCRS comprised a minimal number of reliable chemotherapy-responsive genes; the expression of which could be measured using real time-polymerase chain reaction (RT-PCR) in samples obtained by fine needle aspiration. This provides a potential avenue to monitor resistance to platinum-based chemotherapy in real-time for OvCa patients that respond to initial platinum-based chemotherapy in clinical practice, and redirect patients with chemoresistance to more appropriate therapies in time and avoid unnecessary chemotherapy treatment.

Accumulating evidence suggests that an interplay exists between EMT and chemotherapeutic response in OvCa. However, previous studies investigating the role of EMT in chemoresistance report inconsistent results^24^. For instance, the activation of EMT by the EMT-related signaling pathways, such as endothelin A receptor, p53-mediated apoptosis, epidermal growth factor receptor/signal transducer and activator of transcription 3, transforming growth factor-β and Notch3/ERK signaling pathways^37–39^ or microRNAs, such as microRNA-181a and miR-200 family^40,41^, have been found to be associated with enhanced resistance to platinum-based chemotherapy. By contrast, several studies have indicated that patients or cell lines with mesenchymal phenotypes demonstrated higher sensitivity to cisplatin when compared with the epithelial phenotype^25,42,43^. Therefore, the association between EMT and the chemotherapeutic response was investigated in the current study by constructing an EMT score. The results demonstrated that the EMT score, or the key components of EMT, were all negatively correlated with the IndividCRS across multiple datasets. These results provided further evidence to support the association between EMT and platinum-based chemoresistance.

In conclusion, the present study used an integrated approach involving both OvCa tumors and cell lines, to identify a number of genes that were differentially expressed between platinum-based chemotherapy-resistant samples and sensitive samples. These genes were then used to construct an individualized scoring model consisting of a 16-gene chemotherapy-responsive panel (IndividCRS) for the prediction of response to platinum-based chemotherapy. The ability of the IndividCRS to predict response to platinum-based chemotherapy and outcome was verified in multiple independent datasets deposited by different laboratories with different platform technologies. The universality and simplicity of the IndividCRS make it a promising individualized predictive tool to monitor patient resistance in a timely manner before and after platinum-based chemotherapy treatment, and can be analyzed using RT-PCR in a clinical setting. However, it is should be noted that the IndividCRS was designed to rely on the consistent upregulation of chemo-sensitive genes and the consistent downregulation of chemo-resistant genes. Therefore, in our study, it is merely a reflective signature of the underlying mechanisms of chemo-response, for which the robustness has been validated in many cohorts. To fully understand their role in chemotherapy, functional studies are still required.

## Supplementary information

SUPPLEMENTAL TABLE

SUPPLEMENTAL FIGURES

## References

[1] Torre Lindsey A., Trabert Britton, DeSantis Carol E., Miller Kimberly D., Samimi Goli, Runowicz Carolyn D., Gaudet Mia M., Jemal Ahmedin, Siegel Rebecca L. (2018). Ovarian cancer statistics, 2018. CA: A Cancer Journal for Clinicians.

[2] Doubeni CA, Doubeni AR, Myers AE (2016). Diagnosis and management of ovarian cancer. Am. Fam. physician.

[3] Matulonis UA (2016). Ovarian cancer. Nat. Rev. Dis. Prim..

[4] Kang J, D’Andrea AD, Kozono D (2012). A DNA repair pathway-focused score for prediction of outcomes in ovarian cancer treated with platinum-based chemotherapy. J. Natl. Cancer Inst..

[5] Cancer Genome Atlas Research, N. (2011). Integrated genomic analyses of ovarian carcinoma. Nature.

[6] Blagden SP (2015). Harnessing pandemonium: the clinical implications of tumor heterogeneity in ovarian. Cancer Front. Oncol..

[7] Hartmann LC (2005). Gene expression profiles predict early relapse in ovarian cancer after platinum-paclitaxel chemotherapy. Clin. Cancer Res.

[8] Sabatier R (2011). A seven-gene prognostic model for platinum-treated ovarian carcinomas. Br. J. cancer.

[9] Jazaeri AA (2005). Gene expression profiles associated with response to chemotherapy in epithelial ovarian cancers. Clin. Cancer Res.

[10] Ferriss JS (2012). Multi-gene expression predictors of single drug responses to adjuvant chemotherapy in ovarian carcinoma: predicting platinum resistance. PLoS ONE.

[11] Marchini S (2013). Resistance to platinum-based chemotherapy is associated with epithelial to mesenchymal transition in epithelial ovarian cancer. Eur. J. cancer.

[12] Matondo A (2017). The prognostic 97 chemoresponse gene signature in ovarian cancer. Sci. Rep..

[13] Gonzalez Bosquet J (2016). Prediction of chemo-response in serous ovarian cancer. Mol. Cancer.

[14] Zhou M (2016). Comprehensive analysis of lncRNA expression profiles reveals a novel lncRNA signature to discriminate nonequivalent outcomes in patients with ovarian cancer. Oncotarget.

[15] Zhou M, Zhang Z, Zhao H, Bao S, Sun J (2018). A novel lncRNA-focus expression signature for survival prediction in endometrial carcinoma. BMC Cancer.

[16] Zhou M (2016). Characterization of long non-coding RNA-associated ceRNA network to reveal potential prognostic lncRNA biomarkers in human ovarian cancer. Oncotarget.

[17] Li B, Cui Y, Diehn M, Li R (2017). Development and validation of an individualized immune prognostic signature in early-stage nonsquamous non-small cell lung cancer. JAMA Oncol..

[18] Lloyd KL, Cree IA, Savage RS (2015). Prediction of resistance to chemotherapy in ovarian cancer: a systematic review. BMC Cancer.

[19] Hao D (2017). Integrated analysis reveals tubal- and ovarian-originated serous ovarian cancer and predicts differential therapeutic responses. Clin. Cancer Res.

[20] Konstantinopoulos PA, Ceccaldi R, Shapiro GI, D’Andrea AD (2015). Homologous recombination deficiency: exploiting the fundamental vulnerability of ovarian cancer. Cancer Disco..

[21] Lu J, Wu D, Li C, Zhou M, Hao D (2014). Correlation between gene expression and mutator phenotype predicts homologous recombination deficiency and outcome in ovarian cancer. J. Mol. Med (Berl.).

[22] Ledermann JA, Drew Y, Kristeleit RS (2016). Homologous recombination deficiency and ovarian cancer. Eur. J. cancer.

[23] Li M (2009). Integrated analysis of DNA methylation and gene expression reveals specific signaling pathways associated with platinum resistance in ovarian cancer. BMC Med. genomics.

[24] Klymenko Yuliya, Kim Oleg, Stack M. (2017). Complex Determinants of Epithelial: Mesenchymal Phenotypic Plasticity in Ovarian Cancer. Cancers.

[25] Tan TZ (2014). Epithelial-mesenchymal transition spectrum quantification and its efficacy in deciphering survival and drug responses of cancer patients. EMBO Mol. Med..

[26] Zhang P, Sun Y, Ma L (2015). ZEB1: at the crossroads of epithelial-mesenchymal transition, metastasis and therapy resistance. Cell cycle.

[27] Kiraly O, Gong G, Olipitz W, Muthupalani S, Engelward BP (2015). Inflammation-induced cell proliferation potentiates DNA damage-induced mutations in vivo. PLoS Genet..

[28] Zhang Jun, Dai Qun, Park Dongkyoo, Deng Xingming (2016). Targeting DNA Replication Stress for Cancer Therapy. Genes.

[29] Sun, X. et al. Ki-67 Contributes to normal cell cycle progression and inactive X heterochromatin in p21 checkpoint-proficient human cells. *Mol. Cell. Biol.* **37**, 10.1128/MCB.00569-16 (2017). 10.1128/MCB.00569-16 PMC5559680 28630280

[30] Kawanishi M (2018). Expression of UCP2 is associated with sensitivity to platinum-based chemotherapy for ovarian serous carcinoma. Oncol. Lett..

[31] Odunsi K (2012). Elevated expression of the serine-arginine protein kinase 1 gene in ovarian cancer and its role in Cisplatin cytotoxicity in vitro. PLoS ONE.

[32] L’Esperance S, Bachvarova M, Tetu B, Mes-Masson AM, Bachvarov D (2008). Global gene expression analysis of early response to chemotherapy treatment in ovarian cancer spheroids. BMC Genomics.

[33] Johannsson OT, Ranstam J, Borg A, Olsson H (1998). Survival of BRCA1 breast and ovarian cancer patients: a population-based study from southern Sweden. J. Clin. Oncol..

[34] Yang D (2011). Association of BRCA1 and BRCA2 mutations with survival, chemotherapy sensitivity, and gene mutator phenotype in patients with ovarian cancer. JAMA.

[35] Pothuri B (2013). BRCA1- and BRCA2-related mutations: therapeutic implications in ovarian cancer. Ann. Oncol.: Off. J. Eur. Soc. Med. Oncol..

[36] Christie EL, Bowtell DDL (2017). Acquired chemotherapy resistance in ovarian cancer. Ann. Oncol.: Off. J. Eur. Soc. Med. Oncol..

[37] Deng J (2016). Targeting epithelial-mesenchymal transition and cancer stem cells for chemoresistant ovarian cancer. Oncotarget.

[38] Rosano L (2011). Acquisition of chemoresistance and EMT phenotype is linked with activation of the endothelin A receptor pathway in ovarian carcinoma cells. Clin. Cancer Res.

[39] Kurrey NK (2009). Snail and slug mediate radioresistance and chemoresistance by antagonizing p53-mediated apoptosis and acquiring a stem-like phenotype in ovarian cancer cells. Stem cells.

[40] Park SM, Gaur AB, Lengyel E, Peter ME (2008). The miR-200 family determines the epithelial phenotype of cancer cells by targeting the E-cadherin repressors ZEB1 and ZEB2. Genes Dev..

[41] Parikh A (2014). microRNA-181a has a critical role in ovarian cancer progression through the regulation of the epithelial–mesenchymal transition. Nat. Commun..

[42] Latifi A (2012). Isolation and characterization of tumor cells from the ascites of ovarian cancer patients: molecular phenotype of chemoresistant ovarian tumors. PLoS ONE.

[43] Miow QH (2015). Epithelial-mesenchymal status renders differential responses to cisplatin in ovarian cancer. Oncogene.

