# Supplementary material for: Large-scale integrated analysis of ovarian cancer tumors and cell lines identifies an individualized gene expression signature for predicting response to platinum-based chemotherapy
Source: Cell Death Dis. 2019 Sep 10;10(9):661. doi: 10.1038/s41419-019-1874-9 (PMC6737147; doi:10.1038/s41419-019-1874-9)
Supplement: Supplementary file 2 — SUPPLEMENTAL FIGURES [file 41419_2019_1874_MOESM2_ESM.docx]

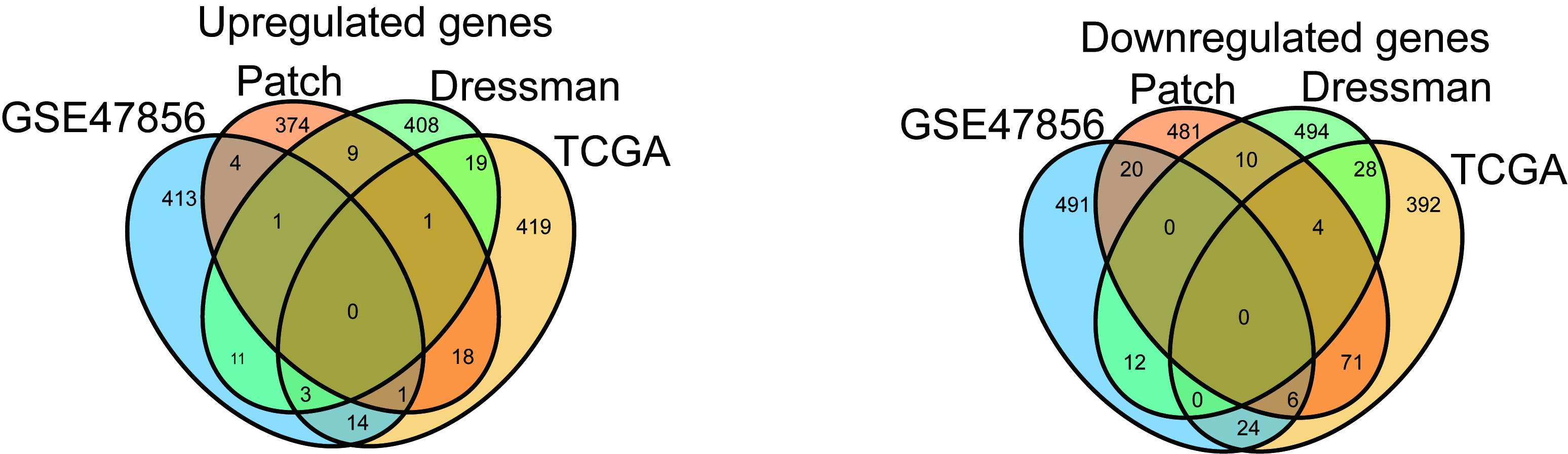


Figure S1. Venn diagram showing the number of differentially expressed genes between resistant samples and sensitive samples across four datasets in the meta-discovery dataset.


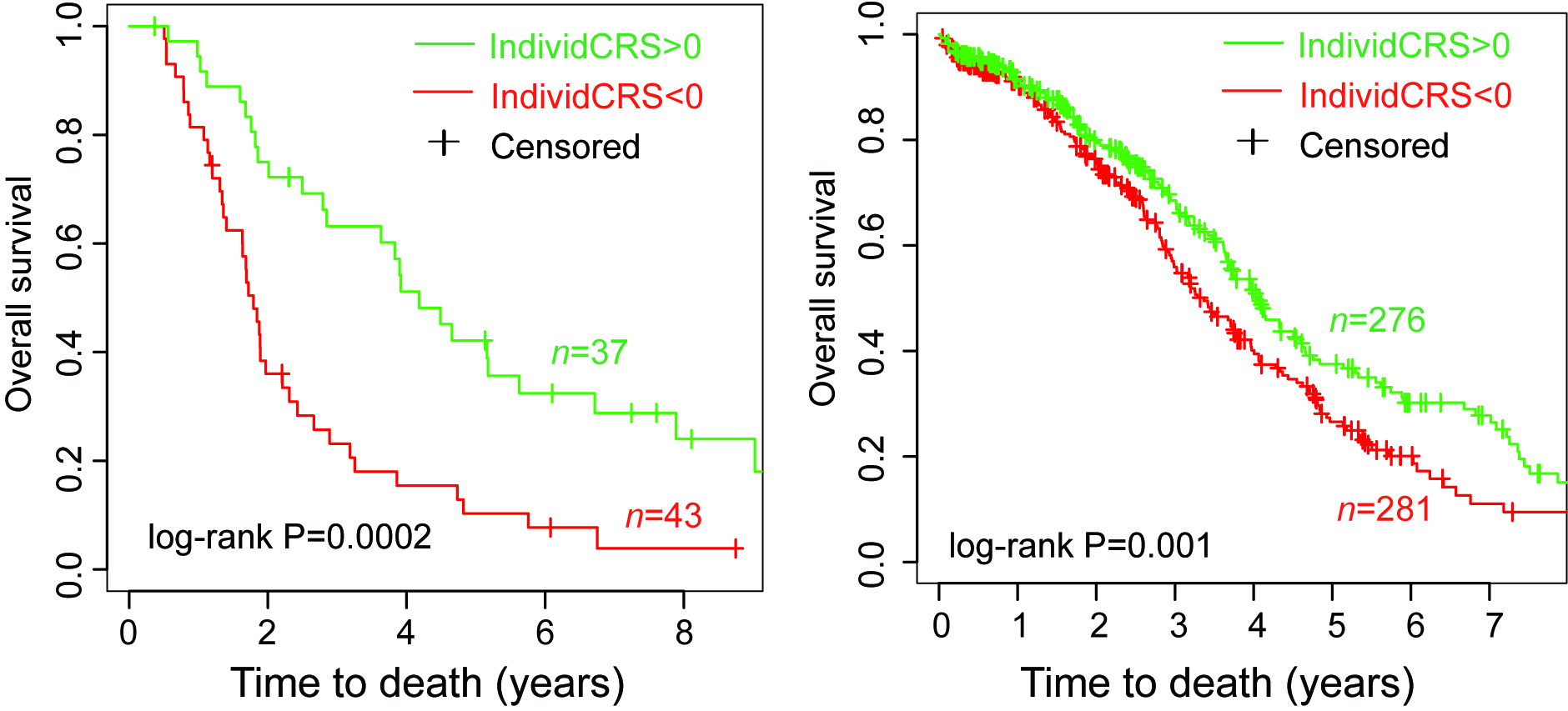


Figure S2. Kaplan-Meier curves of OS for all patients divided by the IndividCRS in the Patch and TCGA datasets.


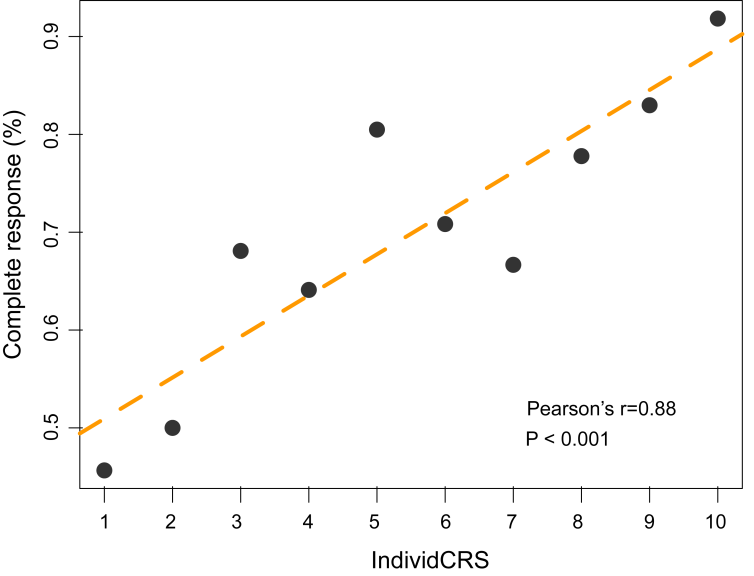


Figure S3. Correlation of between the IndividCRS and the likelihood of complete response (CR)


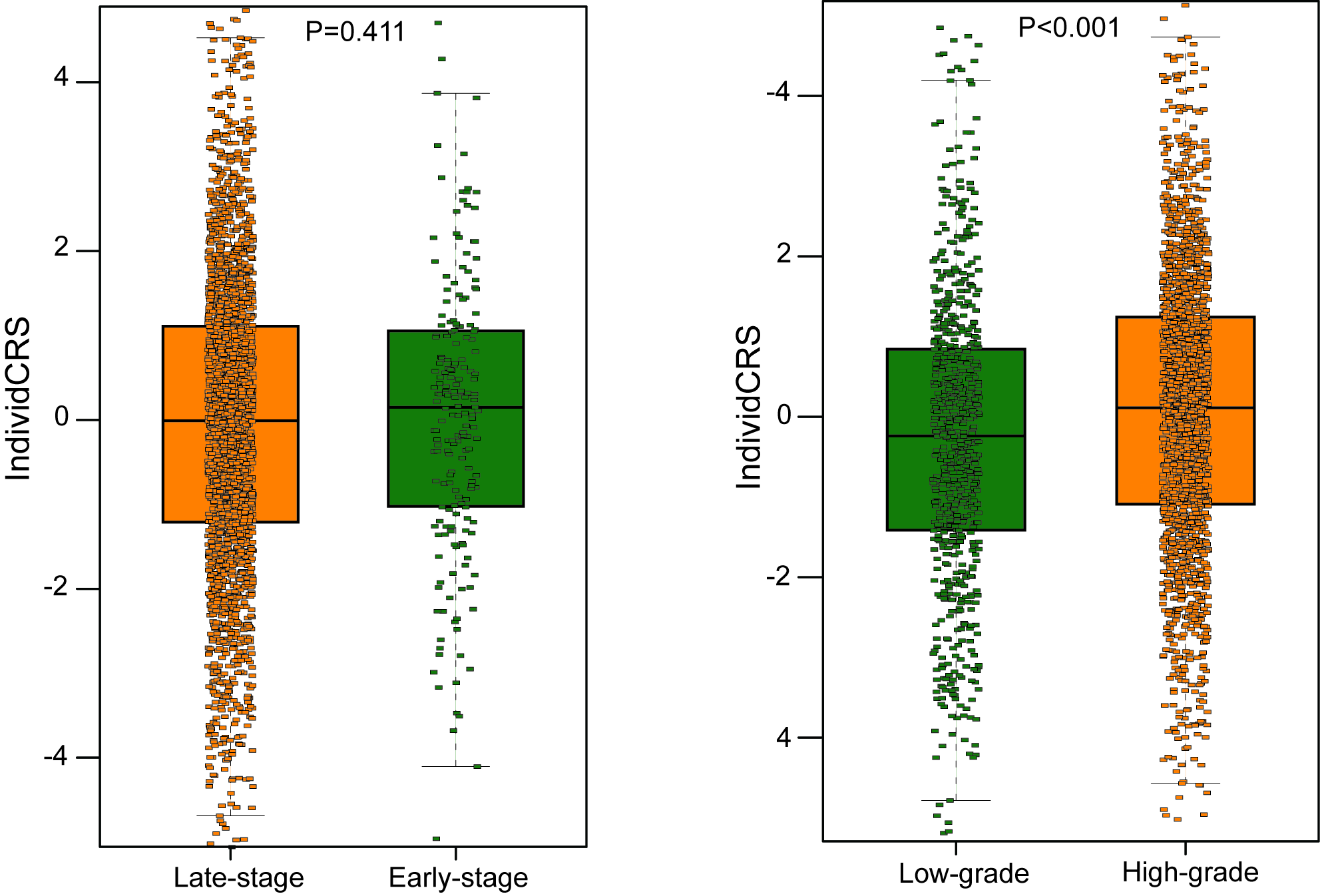


Figure S4. Associations of the IndividCRS with stage and grade. Significance was determined using Mann-Whitney U test.


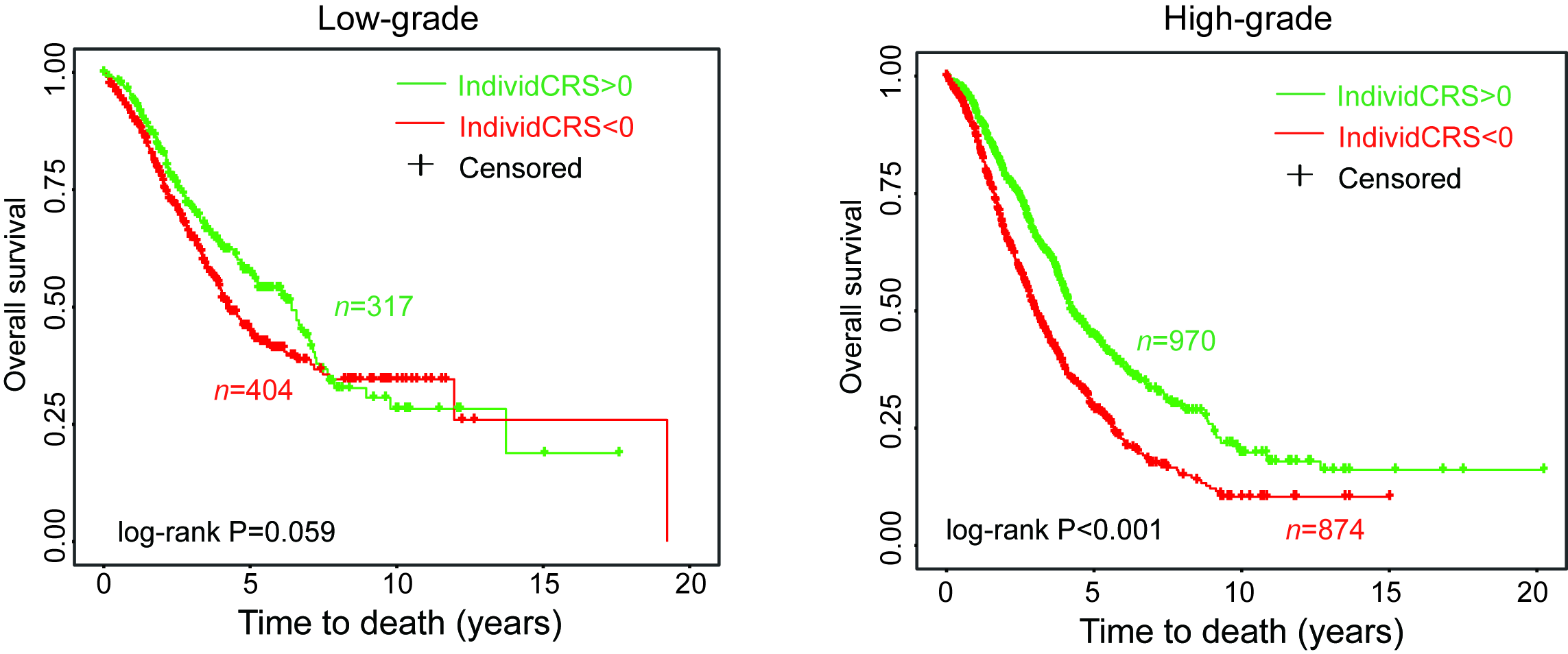


Figure S5. Kaplan-Meier curves of OS for all patients with low grade or high grade divided by the IndividCRS in 21 patient datasets
